# World Allergy Organization Journal: the Editors Look Back at 2016

**DOI:** 10.1186/s40413-017-0140-9

**Published:** 2017-01-23

**Authors:** Erika Jensen-Jarolim, Alessandro Fiocchi

**Affiliations:** 1 Institute of Pathophysiology and Allergy Research, Center of Pathophysiology, Infectiology and Immunology, Medical University of Vienna, Vienna, Austria; 2 Comparative Medicine, The Interuniversity Messerli Research Institute of the University of Veterinary Medicine Vienna, Medical University of Vienna and University of Vienna, Vienna, Austria; 3 Pediatric Hospital Bambino Gesù in Rome, Vatican City, Italy

**Keywords:** Debates in Allergy Medicine, WAO Journal, World Allergy Organization Journal

As the Editors-in-Chief of the official journal of the World Allergy Organization﻿,﻿ we look back at the year 2016 with satisfaction (Fig. 1). The most important themes in allergy were addressed in great part by highly accessed articles. These themes encompass important areas from the new definition of the basics in allergy, from rare clinical cases [1–3] to novel hot topics such as allergy to vaccines [4]. Let us walk together through the past year of the World Allergy Organization Journal (WAO Journal).

**Fig. 1:**
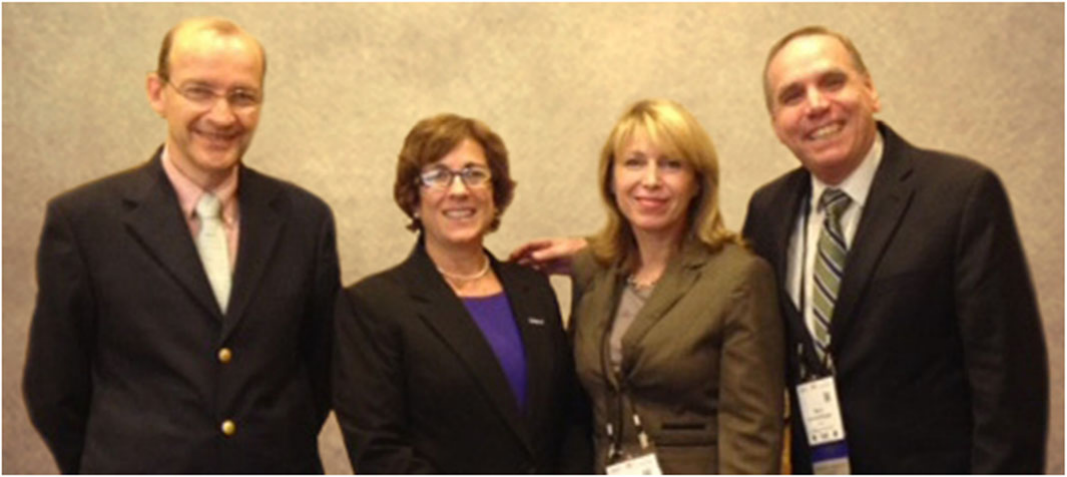
From left: Alessandro Fiocchi, WAO Journal Editor-in-Chief; Sofia Dorsano, WAO Journal Managing Editor; Erika Jensen-Jarolim, MD, WAO Journal Editor-in-Chief; Mario Sánchez-Borges, MD, President, World Allergy Organization, 2016–2017

## Efforts to harmonize the definitions of clinical allergy

With global themes it is important to harmonize language and definitions so that allergic disease classifications can be made universally around the world and communicated without doubt [5]. A similarly important tool is clinical diagnostics, with the skin prick test being an important global diagnostic instrument. In an international survey on skin patch testing a lack of homogeneity was found in an international collaborative, notably with a strong contribution by the Junior Members Group of the World Allergy Organization (WAO) [6]. Allergy is the only discipline with an intrinsically high risk of diagnostic testing as well as of therapeutic intervention by allergen immunotherapy in hypersensitive patients. An international group of experts from different continents evaluated the associated risks and proposed optimal safety measures of a high practical value for allergists and other doctors caring for allergic patients [7]. While allergen immunotherapy is well accepted in allergic rhinitis and asthma, its applicability in atopic patients with eczema is still discussed, and this topic was taken up in two opposing articles within the *Debates in Allergy Medicine* series [8, 9]. The high access numbers for those articles strongly illustrate the interest in this topic among the readership.

## Respiratory allergies deserve global attention

Interestingly, also in respiratory disease the severity of symptoms prompts patients to seek medical advice. This was shown in a Bulgarian study on allergic rhinitis, based on the ARIA guidelines, where more pediatric and adult patients with severe nasal congestion visited physicians [10]. Asthma was addressed in several mega-analyses this year, highlighting novel aspects. An international “manifesto” supported by Interasma, WAO, ARIA and GA2LEN pointed out that the involvement of the small airways should be specifically assessed in asthmatics and COPD, prompting personalized medicine approaches [11]. A European and Canadian mega-survey of 75,335 households revealed that 81% of asthmatics perceived their asthma as well controlled, while still 50% reported acute treatment, implying a discrepancy between perception and actual asthma control [12]. By comparison, in another study approximately one-third of children experienced severe asthma attacks once a year [13]. In the latter study, 734,114 children were analysed, revealing that asthma-related hospitalizations occurred more at younger ages. These numbers underline the ongoing need to improve asthma control by targeted approaches. In this context, an update on inhaled corticosteroids for children with asthma as well as a review on the use of Tiotropium were most appropriate [14, 15], along with a discussion on potential cellular biomarkers which could improve patient stratification [16, 17]. It is important to note that mechanistic basic research also contributed to improved understanding on lung diseases, as discussed in the report on an increased number of T regulatory cells in COPD [18]. Besides environmental concerns, indoor pollution should also be a target for action in the future, and allergen contamination may include monitoring of molecular allergens [19].

## Food allergy – addressing a rising concern

Food allergies are of increasing importance. Among them, milk allergy is a specific concern in children. Besides IgE-mediated, the importance of non-IgE mediated cow’s milk allergy has gained tremendous attention and the urgent need for evidence-based criteria on it was articulated in a call for an update on the DRACMA guidelines [20]. Importantly, current substitution strategies were discussed in this constantly changing field, accompanied by thoughts on food processing methods. The role of baked egg and milk on tolerance development in children [21] also was discussed, as well as the chance to predict the course of egg allergy by provocation tests with heated egg [22]. This topic yielded a hot debate in the *Debates in Allergy Medicine* series [23, 24]. In the middle of the complex clinics of food-associated conditions, the phenotypic diversity of eosinophilic esophagitis (EoE) was addressed with less consequent association to food adverse reactions, but classified as a Th2 disease [25]. The authors emphasized that animal models will be needed to study and understand the different phenotypes in EoE.

## Can we prevent allergies in the future?

Considering their global increase, prevention of allergies remains the “Holy Grail” in our field as well as a hope for the future. Within the series of Guidelines on Allergic Disease Prevention (GLAD-P), two uttermost current topics were addressed in 2016, rendering the following current recommendations: (1) Currently no evidence could be found for Vitamin D supplementation to be effective against allergies in children, neither in pregnant, nor in breastfeeding, women [26]; (2) Prebiotics supplementation should be used in non-exclusively breastfed infants, while – at this time – no recommendation was given for pregnancy or for breastfeeding mothers [27]; (3) a Letter to the Editor claimed the current usage of probiotics was too optimistic, considering the “lack of clear evidence of efficacy of probiotic supplementation in the primary prevention of allergies” [28]. These official statements are important as prebiotics and probiotics increasingly enter the market, due to the suspected potential benefits for children and in atopic eczema [29]. In fact, an Italian consensus document states that for many trendy measures only little if all evidence can be found, such as the use of partially or extensively hydrolyzed formulas, or for a series of dietary supplementations including polyunsaturated fatty acids, vitamins or minerals [30]. A high level of hygiene may be especially detrimental for allergies. On the other hand, poor hygiene in children in a tropical region, assessed by parasite infestations and birth order, rendered either a hypo-responsive or Th2 phenotype, subsequently causing distinct effects on immunologically mediated diseases [31]. This pointed out that there are “particularities of allergy in the tropics” as compared to temperate climate zones, where imminent mite, helminthic and insect sting exposures significantly affect the outcome and management of allergies [32].

Overall, the work published in 2016 in the WAO Journal reconfirmed that the constant global exchange and collaboration of allergy experts across international borders leads to novel recommendations and guidelines and contributes to an overall harmonization. We consider this important in the increasingly fast pace of change in medicine where evidence-based results have to be weighed out against market strategies for the best care of our patients.

We are looking forward to receiving exciting contributions again in 2017!

Erika Jensen-Jarolim, MD and Alessandro Fiocchi, MD

Vienna, Austria, and Vatican City, Italy

December 2016

## Abbreviations

EoE: Eosinophilic Esophagitis
